# Autofluorescence-based high-throughput isolation of nonbleaching *Cyanidioschyzon merolae* strains under nitrogen-depletion

**DOI:** 10.3389/fpls.2022.1036839

**Published:** 2022-12-14

**Authors:** Nozomi Takeue, Ayaka Kuroyama, Yoshiharu Hayashi, Kan Tanaka, Sousuke Imamura

**Affiliations:** ^1^ School of Life Science and Technology, Tokyo Institute of Technology, Midori-ku, Yokohama, Japan; ^2^ Laboratory for Chemistry and Life Science, Institute of Innovative Research, Tokyo Institute of Technology, Midori-ku, Yokohama, Japan; ^3^ Product and Business Planning Section, Planning and Marketing Department, Life Science Business Division, Medical Business Group, Sony Corporation, Nishi-ku, Yokohama, Japan; ^4^ Space Environment and Energy Laboratories, Nippon Telegraph and Telephone Corporation, Musashino-shi, Tokyo, Japan

**Keywords:** nonbleaching strain, phycocyanin, flow cytometer, nitrogen-depletion, red alga

## Abstract

Photosynthetic organisms maintain optimum levels of photosynthetic pigments in response to environmental changes to adapt to the conditions. The identification of cyanobacteria strains that alleviate bleaching has revealed genes that regulate levels of phycobilisome, the main light-harvesting complex. In contrast, the mechanisms of pigment degradation in algae remain unclear, as no nonbleaching strains have previously been isolated. To address this issue, this study attempted to isolate nonbleaching strains of the unicellular red alga *Cyanidioschyzon merolae* after exposure to nitrogen (N)-depletion based on autofluorescence information. After four weeks under N-depletion, 13 cells from 500,000 cells with almost identical pre- and post-depletion chlorophyll a (Chl a) and/or phycocyanin autofluorescence intensities were identified. These nonbleaching candidate strains were sorted *via* a cell sorter, isolated on solid medium, and their post-N-depletion Chl a and phycocyanin levels were analyzed. Chl a levels of these nonbleaching candidate strains were lower at 1–4 weeks of N-depletion similar to the control strains, however, their phycocyanin levels were unchanged. Thus, we successfully isolated nonbleaching *C. merolae* strains in which phycocyanin was not degraded under N-depletion, *via* autofluorescence spectroscopy and cell sorting. This versatile method will help to elucidate the mechanisms regulating pigments in microalgae.

## Introduction

Photosynthesis depends on photosynthetic pigments that absorb light and transfer their excitation energy to the photosynthesis reaction center. Photosynthetic-pigment levels vary with environmental conditions, with nitrogen (N)-depletion being the most studied external cue (Allen and Smith, 1969; Görl et al., 1998). N-depletion degrades photosynthetic pigments, altering the green color of algal cells and plant leaves to yellow-green. This response prevents excessive light energy absorption and allows intracellular N to be recycled for their growth (Collier and Grossman, 1992; Görl et al., 1998).

Photosynthetic pigments captures the light energy necessary for photosynthesis. In cyanobacteria, red algae, and glaucophytes, phycobilisomes are the main light-harvesting complex and contain the photosynthetic pigments, phycobilins, in phycobiliproteins including phycocyanin and allophycocyanin, the component of phycobilisome (Puzorjov and McCormick, 2020). Photosynthetic pigments and proteins containing pigments have specific light absorption and fluorescence maxima. For instance, phycocyanin excitation is at 615–635 nm, and emission at 635–650 nm, and Chl a excitation is at 430 and 660 nm, and emission at 665–675 nm (Butler, 1966; Chen et al., 2015; Elanskaya et al., 2018).

The traditional method of identifying factors regulating photosynthetic pigments involves culturing many candidate strains on solid media, isolating those in which the pigments are not degraded, and identifying the causal genes (Collier and Grossman, 1994; Karradt et al., 2008; Krauspe et al., 2021). Most knowledge about pigment degradation, obtained by studying cyanobacteria, has revealed the causative genes regulating phycobilisome levels in cyanobacteria. In the cyanobacterium *Synechococcus* sp. PCC 7942, a regulator involved in phycobilisome degradation, NblA, was identified using nonbleaching mutants (created using N-methyl-N’-nitro-N-nitrosoguanidine), to elucidate the molecular mechanism of phycobilisome degradation (Collier and Grossman, 1994). NblA expression is enhanced under N-depletion: it first binds to the α-subunit of the phycobiliprotein CpcA; a chaperone partner of the Clp protease HSP100, ClpC, then binds to NblA, in an ATP-dependent manner, bringing ClpC and the phycobiliprotein subunits into proximity, promoting phycobilisome degradation (Baier et al., 2001; Karradt et al., 2008). NblB, an NblA homolog, is directly involved in phycocyanin degradation in an NblA-dependent manner: degradation of the phycocyanin hexamer and trimer in the phycobilisome rod portion by NblA results in the formation of an NblA-phycocyanin monomer, which is degraded by NblB (Li and Sherman, 2002; Levi et al., 2018). NblD, which specifically binds to the phycocyanin beta subunit (CpcB) of phycobilisomes, is another critical regulator of phycobilisome turnover: N-depletion induced *nblD*-knockout strain pigmentation was only marginally reduced, similar to *nblA*-knockout strains (Krauspe et al., 2021).

In contrast, the regulators and mechanisms involved in photosynthetic-pigment degradation in algae are very limited. For example, in the unicellular red alga *Cyanidioschyzon merolae* (Kuroiwa, 1998), it has been reported that RNA polymerase sigma factors SIG2 and SIG4 are positively and negatively involved in phycobilisome content, respectively (Fujii et al., 2013; Fujii et al., 2015). To reveal the overall pigment degradation mechanism in algae, isolation of nonbleaching mutants is a useful approach like done in cyanobacteria, however, it requires a lot of time and effort since many colonies must be isolated on the plates, and each strain’s phenotype must be observed. Furthermore, it’s also hard to identify gene(s) that causes the phenotype after isolation of the mutant among the mutated cell population. Therefore, a simple and high-throughput method to isolate nonbleaching mutants using non-mutagenesis cells that can adapt generally to algae is eagerly awaited. To address this issue, in this study, we present a novel high-throughput method using autofluorescence (spontaneous light emission by biological structures when they absorb light) without any mutagenesis treatment to isolate photosynthetic-pigment-degradation mutants, using *C. merolae*. We hypothesized that this method would reveal cellular changes, such as photosynthetic-pigment degradation, which occur under N-depletion. *Via* spectral cell analysis and cell sorting, we successfully isolated *C. merolae* strains that are nonbleaching under N-depletion.

## Materials and methods

### Strains and growth conditions

*Cyanidioschyzon merolae* 10D wild-type, nonbleaching, and bleaching strains were cultured at 40°C under continuous white light (50 μmol m^−2^ s^−1^) in liquid MA2 medium at pH 2.5 (Imamura et al., 2010) bubbled with air supplemented with 2% (v/v) CO_2_. For cultivation in 96 and 24 well plates, the cells were incubated under continuous white light (20 μmol m^−2^ s^−1^) and 5% (v/v) CO_2_. N-depletion was as described in Imamura et al. (2009).

### Measurement of cell autofluorescence

The cell cultures were passed through a 5 µm filter (pluriSelect Life Science UG & Co. KG) to remove aggregated *C. merolae* cells. This step is just in case since large aggregated cells cause erroneous data and machine trouble. Actually, no cells trapped with the filter were visibly observed. A portion of the filtrate was aliquoted onto a OneCell counter (OneCell, Tokyo, Japan), and the cells were counted under a microscope (NIKON CORPORATION, Tokyo, Japan) to determine cell concentration. The cell cultures were diluted to approximately 1×10^7^ cells/mL, and its autofluorescence was measured using an SA3800 Spectral Cell Analyzer (SONY, Tokyo, Japan). SA3800 Spectral Cell Analyzer is a spectral flow cytometer that detects emission fluorescence from individual cells with prisms and acquires the full fluorescence spectra ranging from 500 nm to 800 nm with a photomultiplier (Futamura et al., 2015). Spectral flow cytometry is one of the approaches to measure algal autofluorescence (Dashkova et al., 2016). SA3800 Spectral Cell Analyzer is not a flow cytometer equipped with a cell sorter, thus, is not able to separate and collect cells of interest. The diluted sample (2 mL) was dispensed into a nonsterile 5-mL tube (Round Bottom Polypropylene Test Tube without Cap, 12 × 75 mm, Fisher Scientific, Loughborough, UK), and placed in the Spectral Cell Analyzer. Excitation lasers at 488 nm and 638 nm were used for measurement. Based on the forward-scattered light (FSC) and side-scattered light (SSC) data obtained, scatter plots of SSC area against FSC intensity were generated. The plot reflecting the largest population was selected for further analysis. Based on these plots, scatter plots of FSC intensity against FSC area were generated; plots with a ratio of ca. 1:1 were extracted. These selection processes removed noise other than the measured cells and aggregated cells. For each sample, the extracted plot provides its autofluorescence spectrum. Spectral plots reflect accumulative intensity from every single cell in the cell cultures.

### Cell sorting

Cells with the desired fluorescence intensity were aliquoted using the SH800S Cell Sorter (SONY), using the same excitation lasers that were used to measure autofluorescence. Two bandpass filters, at 665/30 nm and 720/60 nm, were used to distinguish the cells of interest. For cell sorting, we used a 70 µm sorting tip, with sorting mode set to ‘Normal,’ and sample pressure set to 4; sorting was conducted at room temperature (approximately 25°C). Cells were aliquoted into 100 µL of MA2 liquid medium in 96-well plates. Some cells were sorted into 1 mL of MA2 liquid medium in 5 mL tubes. The sorted cells in the 5 mL tubes were again subjected to autofluorescence spectrophotometry (SA3800 Spectral Cell Analyzer) to confirm selection of the desired cells. After culturing the sorted cells, the cell cultures were diluted 10-, 100-, and 1000-fold in MA2 liquid medium, spread on MA2 solid medium (Takemura et al., 2019), and single cells were isolated. Each colony was collected and cultured in a 96-well plate containing 100 µL of MA2 liquid medium per well. Following culturing, Chl a and phycocyanin levels were measured.

### Chl a and phycocyanin measurement

*C. merolae* cultures were prepared at an OD_750_ of 0.2–0.8. OD_750_ was measured using a UV-visible DU730 spectrophotometer (Beckman Coulter, Brea, CA). Absorbance was measured continuously at 700–400 nm, using a U-3900 spectrophotometer (Hitachi, Tokyo, Japan). Aluminum oxide 210-0740 (Hitachi High-Tech Science, Tokyo, Japan) was used as the subwhite plate. From the absorbance values, cellular Chl a and phycocyanin levels were determined, as follows (Arnon et al., 1974):


Chl a (µg/ml)= 14.97  A678 − 0.615  A620

Phycocyanin (µg/ml)= 138.5  A620 − 35.49  A678

## Results

### *C. merolae* autofluorescence under N-depletion

**Figure 1:**
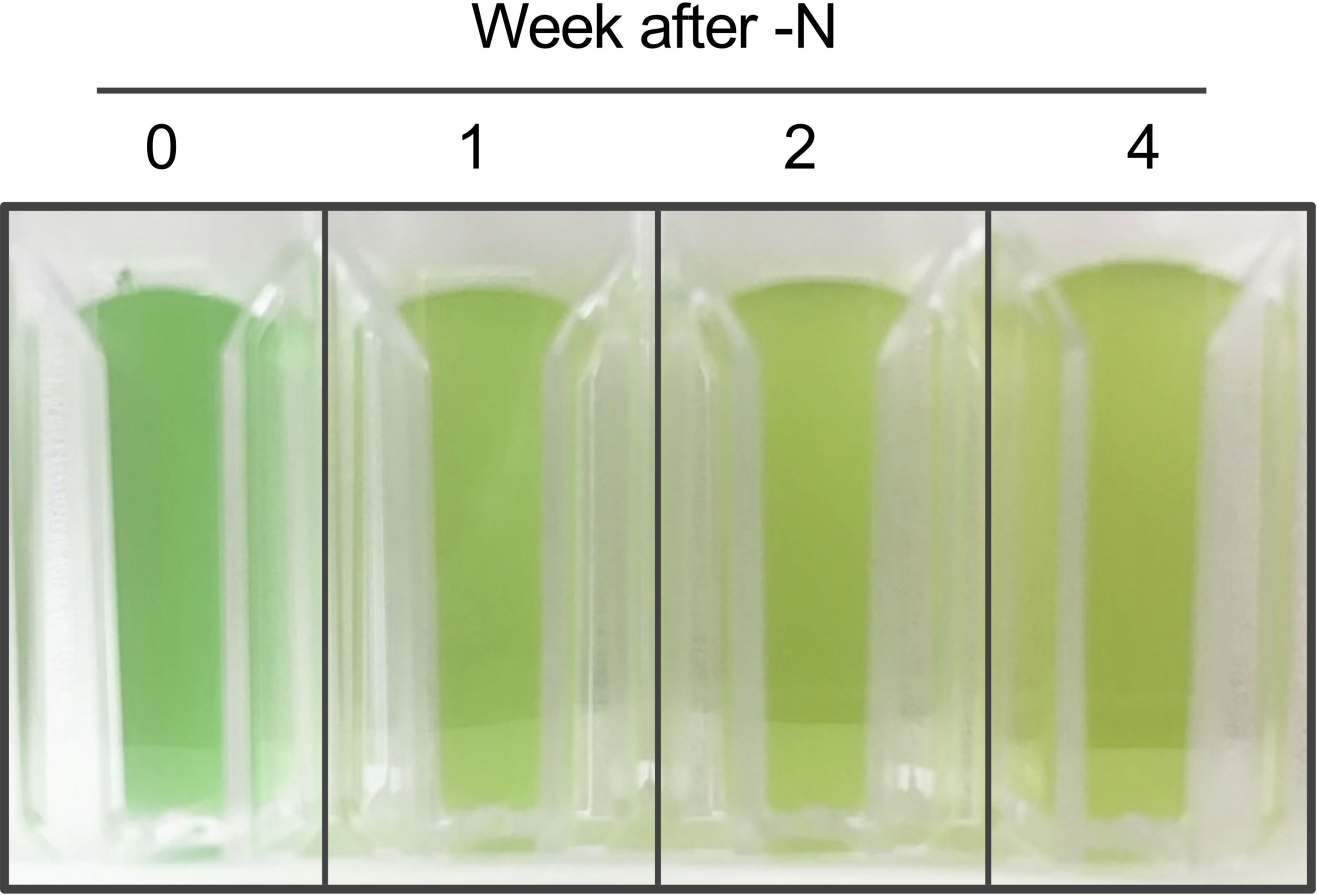
Photograph of *Cyanidioschyzon merolae* culture after N-depletion. Cultures were photographed at the indicated times after N-depletion.

**Figure 2:**
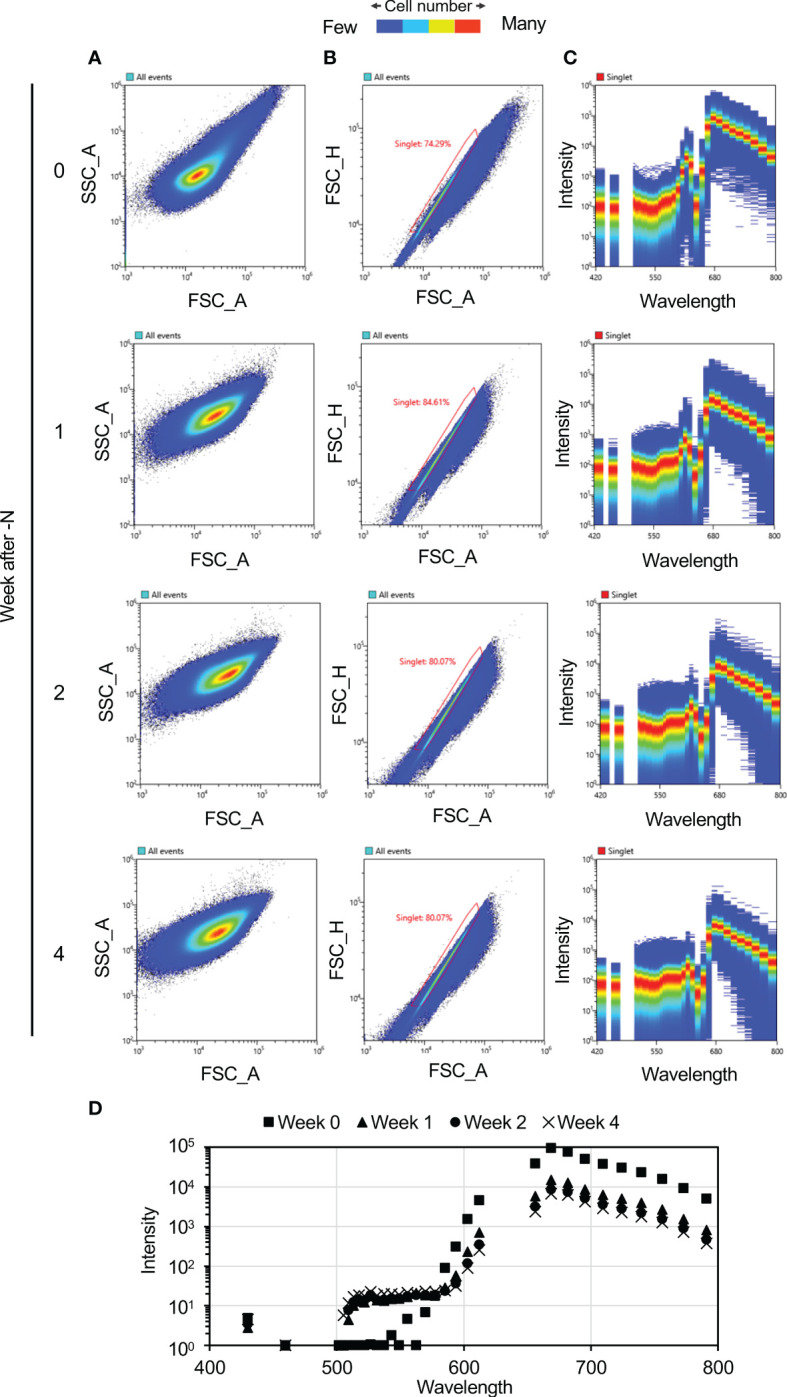
Autofluorescence spectra of the cultured cells. Plots were generated using a Spectral Cell Analyzer SA3800 (SONY). **(A, B)** Scatter plots of *Cyanidioschyzon merolae* cells exposed to N-depletion at the indicated times as weekly intervals using a Spectral Cell Analyzer SA3800. **(A)** Scatter plot showing forward-scatter area (FSC_A) *vs*. side-scatter area (SSC_A). **(B)** Singlet plot showing FSC_A *vs*. forward-scatter height (FSC_H). Single cells were distinguished based on information in the singlets plot. The percentages shown in this panel denote the percentage of singlets among the analyzed cells. **(C)** Fluorescence intensity visualization for each cell. The numbers of cells contributing to each plot, by weeks of N-depletion, are as follows: zero week, 1,857,225; one week, 2,115,228; two weeks, 2,150,198; and four weeks, 2,001,770. **(D)** Average fluorescence intensities of the cells at weekly intervals.

Changes in *C. merolae* cell autofluorescence under N-depletion were measured at weekly intervals. Cell color progressively changed from green to yellow-green under N-depletion, visually confirming bleaching (**Figure 1**). Autofluorescence spectra of each cell were acquired at 420–800 nm after N-depletion. The autofluorescence at 469–500 nm and 617–650 nm was not measured because of spectral attenuation at these wavelengths due to the notch filter. Scatter plots were used to select single-cell spectra (**Figures 2A, B**), eliminating multiple-cell populations. **Figure 2C** shows the autofluorescence and intensity derived from single cells (approximately 2 million cells each time point) at weekly intervals after N-depletion. Fluorescence intensity changed with time under N-depletion (**Figures 2C, D**, **S1A**), primarily increasing at ca. 500–570 nm and decreasing at ca. 680 nm. We note that the fluorescence intensities at ca. 500–570 nm seem to be the same irrespective of the N status since the patterns of red dots seems to be almost the same. However, a clear difference between 0 week and 1–4 weeks after N-depletion can be found, especially the number of blue dots of which intensities are high compared with red dots at ca. 500–570 nm is clearly increased. Therefore, the values of average shown in **Figure 2D** are clearly different before and after N-depletion.

### Isolation of *C. merolae* nonbleaching strains under N-depletion

**Figure 3:**
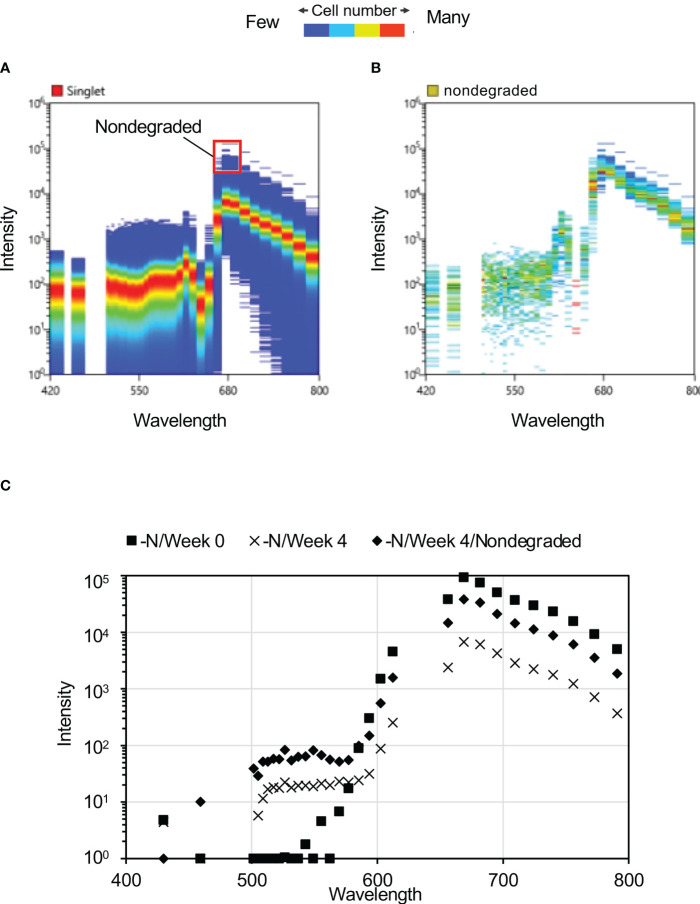
Autofluorescence spectra of cells after four weeks of N-depletion. **(A)** Autofluorescence spectrum derived from a single cell, after four weeks of N-depletion, based on 2,001,770 plots. The “nondegraded” group refers to cells with autofluorescence intensity of ca. 680 nm, where photosynthetic pigment was not substantially reduced by four weeks of N-depletion; it comprises 0.0057% of the total number of cells measured. **(B)** Autofluorescence spectrum of 115 “nondegraded” cells. **(C)** Average autofluorescence intensity of each culture. –N/Week 0 and –N/Week 4: average autofluorescence intensities at each wavelength for whole single cells, after zero and four weeks of N-depletion, respectively. nondegraded: average autofluorescence intensities at each wavelength for the nondegraded group after four weeks of N-depletion.

Maximum reduction in peak autofluorescence at ca. 680 nm was observed after four weeks of N-depletion (**Figure 2**), hence we focused on this exposure duration for further analysis. At four weeks, some of the cells with spectra intensity ca. 680 nm were unaltered by N-depletion, reflecting a lack of pigment degradation (“nondegraded” cells, **Figure 3A**). These nondegraded cells had a maximum mean fluorescence intensity 28.5 times higher than that of the cells with reduced fluorescence intensity. At four weeks, the nondegraded-cell autofluorescence intensities (**Figure 3B**) were clearly higher than those for all other cells (**Figures 3C**, **S1B**) by 6.2-fold at 656 nm (the approximate Chl a autofluorescence), and 5.6-fold at 668 nm (the approximate phycocyanin autofluorescence). The nondegraded group comprised 13 cells from a population of 488,800 single cells derived from 500,000 cells.

**Figure 4:**
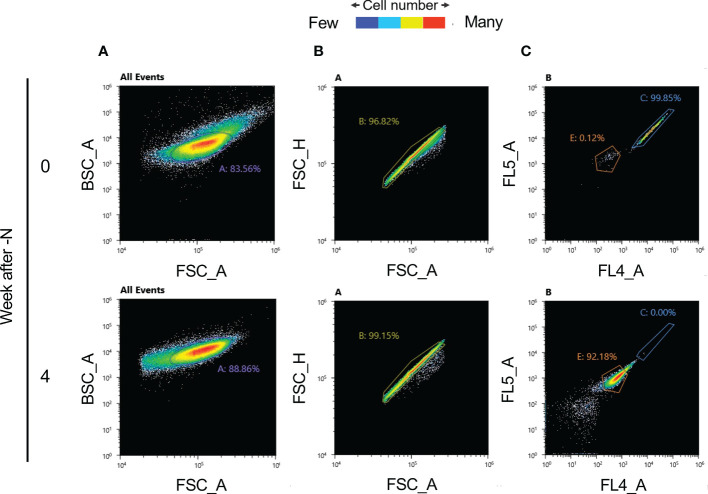
Autofluorescence values and cell isolation before and after N-depletion, obtained using an SH800S Cell Sorter (SONY). **(A)** Back-scatter area (BSC_A) and forward-scatter area (FSC_A) scatter plots for each cell following N-depletion, (100,000 measured cells). The number A denotes the percentage of cells inside the purple circle (gate A). **(B)** FSC_A and FSC_H scatter plot. Scatter plot of forward-scatter area (FSC_A) and forward-scatter height (FSC_H) based on each cell following N-depletion. For the start of N-depletion, there are 83,556 plots; for four weeks of N-depletion, there are 88,856 plots. Gate B (the yellow circle) denotes single cells based on scatter plot information. The number B denotes the percentage of cells inside the yellow circle. For the start of N-depletion, there are 80,903 plots; for four weeks of N-depletion, there are 88,105 plots. **(C)** Autofluorescence scatter plot, for the single cells identified in panel B, at 720 ± 30 nm (FL5 detector) and 665 ± 15 nm (FL4 detector). For the start of N-depletion, there are 80,903 plots; for four weeks of N-depletion, there are 88,105 plots. Gates C (blue circle) and E (orange circle) denote cell populations whose autofluorescence is mostly as expected before N-depletion, or after four weeks of N-depletion, respectively. The numbers C and E denote percentages of cells inside the blue and orange circles, respectively.

After isolating these nondegraded cells using a cell sorter, we attempted to obtain nonbleaching strains without mutagen treatment (**Figures 4A, B**). The excitation fluorescence intensities at 720 ± 30 nm (obtained using an FL5 detector), and at 665 ± 15 nm (using an FL4 detector) decreased substantially with N-depletion (cell group C represents nonbleaching cells and E is the group of bleaching cells; **Figure 4C**). Group C was aliquoted into 96-well plates (100 µl of MA2 medium/well) for culture. However, no cell proliferation was observed. This is probably because it is difficult to culture cells after single-cell aliquoting.

To examine the conditions under which *C. merolae* cells can grow after cell sorting, 50, 100, 500, and 1000 cells of the bleaching candidate strain, and 50 and 100 cells of the nonbleaching candidate strain were aliquoted into each well containing 100 µl of MA2 medium using the cell sorter. The cells can be damaged by the extremely narrow tip diameter during sorting, hence we examined various tip diameters for sorting. The results showed that both the number of cells and tip diameter affected subsequent cell growth (**Figure S2**). We observed growth under the following conditions: for the bleaching strain, >100 cells/well with 70 µm sorting tip and >50 cells/well with 100 µm sorting tip; for the nonbleaching strain, >50 cells/well with 70 µm sorting tip and >100 cells/well with 100 µm sorting tip. Based on these results, the optimal cell sorting conditions were determined to be 100 cells/well for the bleaching strain and 50 cells/well for the nonbleaching strain, using 70 µm sorting tip for both strains.

**Figure 5:**
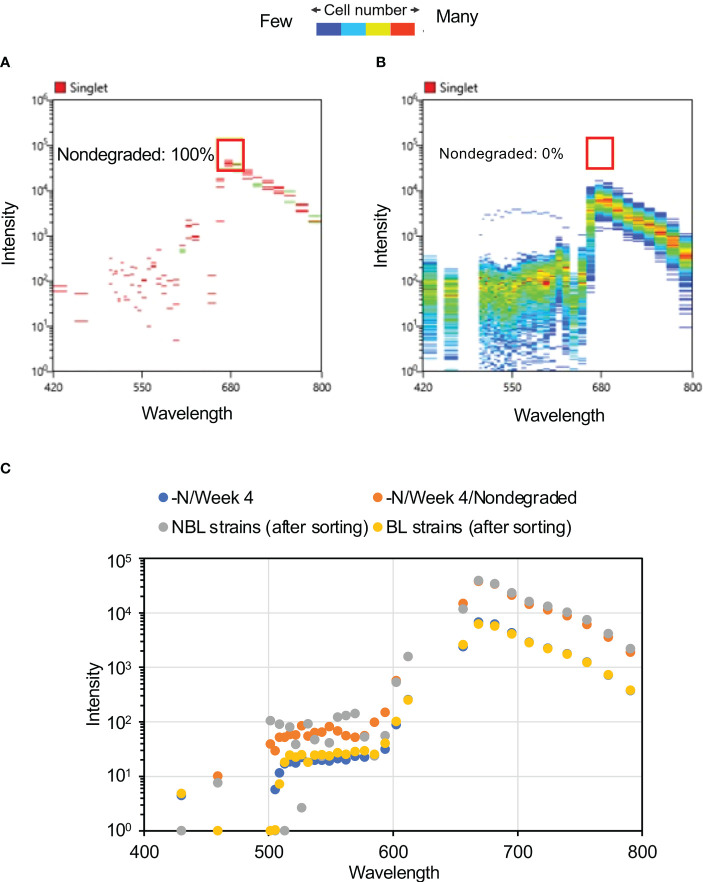
Autofluorescence spectra of candidate nonbleaching cells. **(A)** Autofluorescence spectrum derived from single cells of four nonbleaching candidate strains after four weeks of N-depletion. The red box and number denote area corresponding to the nondegraded group and the proportion falling within the red box. **(B)** Autofluorescence spectrum derived from single cells of 703 bleaching candidates after four weeks of N-depletion. None of these cells shared the same autofluorescence as the nondegraded group. **(C)** Mean autofluorescence intensity of each culture or sorted cell. The post-sorting values for the nonbleaching and bleaching strains are mean values at each of the wavelengths shown in panels **(A)** and **(B)**. The graphs of –N/Week 4 and –N/Week 4/Nondegraded are the same data shown in **Figure 3C**.

Using the optimal sorting conditions, we attempted to isolate nonbleaching and bleaching candidate strains based on the fluorescence intensities of Chl a (720 ± 30 nm) and phycocyanin (665 ± 15 nm) of cells subjected to four weeks of N-depletion. From the 100,000 cells examined, four nonbleaching candidate cells were isolated by this approach (**Figure 4C**). It is conceivable that the four isolated nonbleaching candidate cells were not single isolated cell lines, but groups of sorted cells that might be genetically heterogeneous. To validate the isolation, we measured the autofluorescence spectrum of each cell of the bleaching and nonbleaching strains isolated by the cell sorter: the candidate nonbleaching and bleaching strains showed autofluorescence patterns similar to the nondegraded cells and those exposed to four weeks of N-depletion, respectively. For the four nonbleaching candidate cells, the autofluorescence at ca. 680 nm was almost identical to the nondegraded group’s autofluorescence (**Figures 5A, B**).

**Figures 5C** and **S1C** shows the average fluorescence intensities at the indicated wavelengths for the bleaching and nonbleaching strains. The spectrum of the nonbleaching candidate strain was largely consistent with that of the nondegraded group (**Figure 3C**), whereas that of the bleaching candidate strain was consistent with that of cells grown under N-depletion for 4 weeks (**Figure 5B**). Comparing the four-week-N-depletion pre-sorting and bleaching group post-sorting cells, the correlation coefficient was 0.999 for the nondegraded group and it was 0.997 for the nonbleaching-post-sorting group, demonstrating that the nonbleaching strain’s autofluorescence at the selected wavelengths for Chl a and/or phycocyanin was not reduced by N-depletion. Based on this, nonbleaching and bleaching candidate strains were aliquoted into each well of a 96-well plate under the optimal conditions using a cell sorter, at 600 cells per 12 wells (50 cells per well) for the nonbleaching strain, and 600 cells per six wells (100 cells per well) for the bleaching strain. After growth in each well in normal medium, the cells were plated on solid medium and single cells were isolated as independent strains (**Table 1**).

**Table 1:** Correspondence table of each strain and its origin.

Strain	Origin of well in the 96 plate
NBL-1	C-2
NBL-2	C-2
NBL-3	C-2
NBL-4	C-2
NBL-5	C-2
NBL-6	C-4
NBL-7	C-4
NBL-8	C-4
NBL-9	C-4
NBL-10	C-4
NBL-11	C-5
BL-1	E-1
BL-2	E-1
BL-3	E-3
BL-4	E-3

### Changes in nonbleaching-strain photosynthetic-pigment content under N-depletion

**Figure 6:**
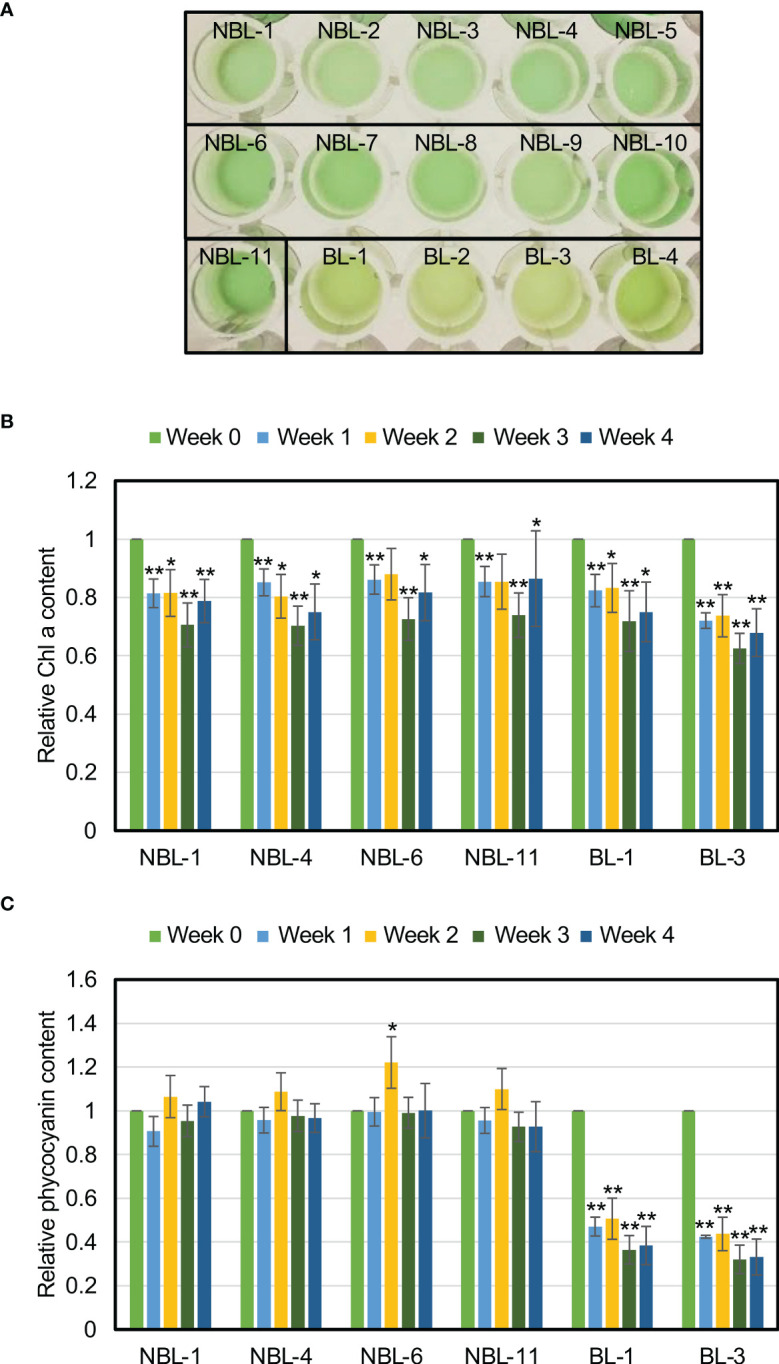
Alleviation of phycocyanin degradation under N-depletion in the nonbleaching strains. **(A)** Nonbleaching and bleaching strain cultures, after one week of N-depletion. **(B)** Chl a levels for the nonbleaching and bleaching strains. Chl a levels in strains NBL-1, NBL-4, NBL-6, NBL-11, BL-1, and BL-3, as a proportion of the value at the start of N-depletion (*t*-tests comparing the pre- and post-N-depletion results for each strain; **p < 0.01; *p < 0.05). **(C)** Phycocyanin levels of representative nonbleaching and control strains. Phycocyanin levels in strains NBL-1, NBL-4, NBL-6, NBL-11, BL-1, and BL-3, as a proportion of the value at the start of N-depletion (*t*-tests comparing the pre- and post-N-depletion results for each strain; **p < 0.01; *p < 0.05).

Among the 84 isolated nonbleaching strains, we selected 11 (NBL-1 to NBL-11) strains as representative strains, and visually checked photosynthetic-pigment degradation after one week of N-depletion (**Figure 6A**). We analyzed four bleaching strains (BL-1 to BL-4) as representative controls. The bleaching phenotype was observed in the bleaching strains, as in the wild-type (**Figure 1**), but not in the nonbleaching strains (**Figure 6A**). The following strains were isolated from the same wells: NBL-1–5; NBL-6–10; BL-1–2; and BL-3–4 (**Table 1**).

We then quantified Chl a and phycocyanin levels under N-depletion for NBL-1, NBL-4, NBL-6, NBL-11, BL-1, and BL-3 (**Figures 6B, C**). In addition to one week after N-depletion the same as in **Figure 6A**, we measured Chl a and phycocyanin levels at two, three, and four weeks after N-depletion due to check the possibility of the growth-phase specific changes of those levels. The Chl a and phycocyanin levels during each weekly measurement are presented as a fraction of their original values (at 0 week). Chl a levels were significantly lower during all measurements under N-depletion, for both the nonbleaching and bleaching strains (**Figure 6B**). Phycocyanin content was significantly lower during measurement for both bleaching strains, but remained unchanged for the nonbleaching strains irrespective the time after N-depletion (**Figure 6C**). This nonbleaching phenotype of NBL strains was observed for at least approximately one year as far as we check until now, indicating the phenotype is genetically stable. These results confirm that nonbleaching under N-depletion is due to the alleviation of phycocyanin degradation.

## Discussion

To elucidate the regulators and mechanisms involved in photosynthetic-pigment degradation in microalgae, we applied a novel method combining a Spectral Cell Analyzer and cell sorting and used autofluorescence intensity as an indicator of photosynthetic-pigment levels. This method enabled successful isolation of nonbleaching *C. merolae* strains from the wild-type without requiring mutagen treatment.

The nonbleaching strains showed reduction in Chl a during N-depletion as the bleaching strains (**Figure 6**). The reduction of Chl levels under N-depletion has been generally observed not only in cyanobacteria and algae but also in land plants to recycle nitrogen that is contained in Chl (Matile et al., 1999; Avila-Ospina et al., 2014). Whereas the nonbleaching strains lacked phycocyanin degradation during N-depletion (**Figure 6**). In cyanobacteria, NblA, NblB, and Clp protease are reported to degrade phycocyanin, and the degradation mechanisms are known. Our study species, *C. merolae*, contained homologs of NblA and ClpC (Matsuzaki et al., 2004), and the nonbleaching strains could have mutations in these factors. *NblA* expression is strongly induced in cyanobacteria during phycocyanin degradation under N-depletion (Collier and Grossman, 1994). In contrast, for *C. merolae*, we have previously found that *NblA* (*CMV052C*) expression is reduced during N-depletion (Fujii et al., 2015). Therefore, both common and distinct mechanisms may contribute to phycocyanin degradation in cyanobacteria and red alga. The other mutation possibility is that genes that involved in regulators of gene expression for PBS level. In *C. merolae*, as mentioned in the Introduction, SIG2 and SIG4 are involved in phycobilisome levels, raising the possibility of mutation in the two genes. Furthermore, it is known that epigenetics play important roles in adaptation to environmental changes, such as light, heat shock, and nutrient conditions (Bacova et al., 2020). So, it raises also the possibility of mutations in genes encoding epigenetic regulators for transcription of *NblA*, *ClpC*, *SIG2*, *SIG4*, and other genes involved in PBS levels. Next-generation genome sequencing of our novel *C. merolae* nonbleaching strains may validate this and help to identify the regulators involved in phycocyanin degradation in microalgae. Spontaneous mutants, as in this study, are likely to have few DNA mutation sites, making it relatively easy to identify the genes responsible for the nonbleaching phenotype. Future studies of these spontaneous mutants will help to elucidate the mechanisms regulating photosynthetic pigments in microalga.

Both nonbleaching and bleaching strains were identified in this study (**Figure 6** and **Table 1**), although it is possible that these strains are derived from the same original cells since they were selected from the same wells during cell sorting. We intend to validate this in future, by sequencing each strain’s genome. The autofluorescence spectrum analyzer could detect 13 nonbleaching cells out of 500,000 (0.0026%) and the cell sorter could detect 4 nonbleaching cells out of 100,000 (0.004%). In contrast, it has been reported that 100 nonbleaching candidate strains were isolated from 200,000 cells (0.05%) by the traditional method of isolation of nonbleaching candidates on solid medium (Collier and Grossman, 1994). The current method reported in this study generates a lower rate of the nonbleaching strains compared to the traditional method. However, the current method can isolate nonbleaching strains at high-throughput with autofluorescence spectrum analyzers and cell sorters which are capable of acquiring or fractionating information on large numbers of cells in a short time (e.g., 5,000 cells are measured per second). Moreover, this method has the advantage of making it possible to isolate nonbleaching cells generated by spontaneous mutation from the cell population without the need for mutagenesis.

The N-depleted cells exhibited change in autofluorescence at ca. 500–570 nm, which is not attributable to Chl a or phycocyanin. This autofluorescence may be due to increased riboflavin, which autofluoresces at 550 nm (Zipfel et al., 2003). Thus, it may also be possible to isolate mutant strains with varying riboflavin levels by using autofluorescence changes at ca. 500–570 nm by following our isolation method for nonbleaching strains from this study.

In conclusion, we were able to establish a novel method by combining autofluorescence spectroscopy and cell sorting to successfully isolate nonbleaching *C. merolae* strains. High-throughput isolation using this method should be possible and easy for unicellular microalgal strains with abnormal photosynthetic-pigment degradation or synthesis. This versatile method may become fundamental in elucidating the mechanisms regulating pigments in microalgae.

## Data availability statement

The original contributions presented in the study are included in the article/**Supplementary Material**. Further inquiries can be directed to the corresponding author.

## Author contributions

SI, AK designed the research. NT, AK and SI conducted the research. NT, AK, YH, KT, and SI analyzed the data. and SI wrote the manuscript. All authors contributed to the article and approved the submitted version.
