# Supplementary figures and images for: Autofluorescence-based high-throughput isolation of nonbleaching Cyanidioschyzon merolae strains under nitrogen-depletion
Source: Front Plant Sci. 2022 Dec 14;13:1036839. doi: 10.3389/fpls.2022.1036839 (PMC9794624; doi:10.3389/fpls.2022.1036839)

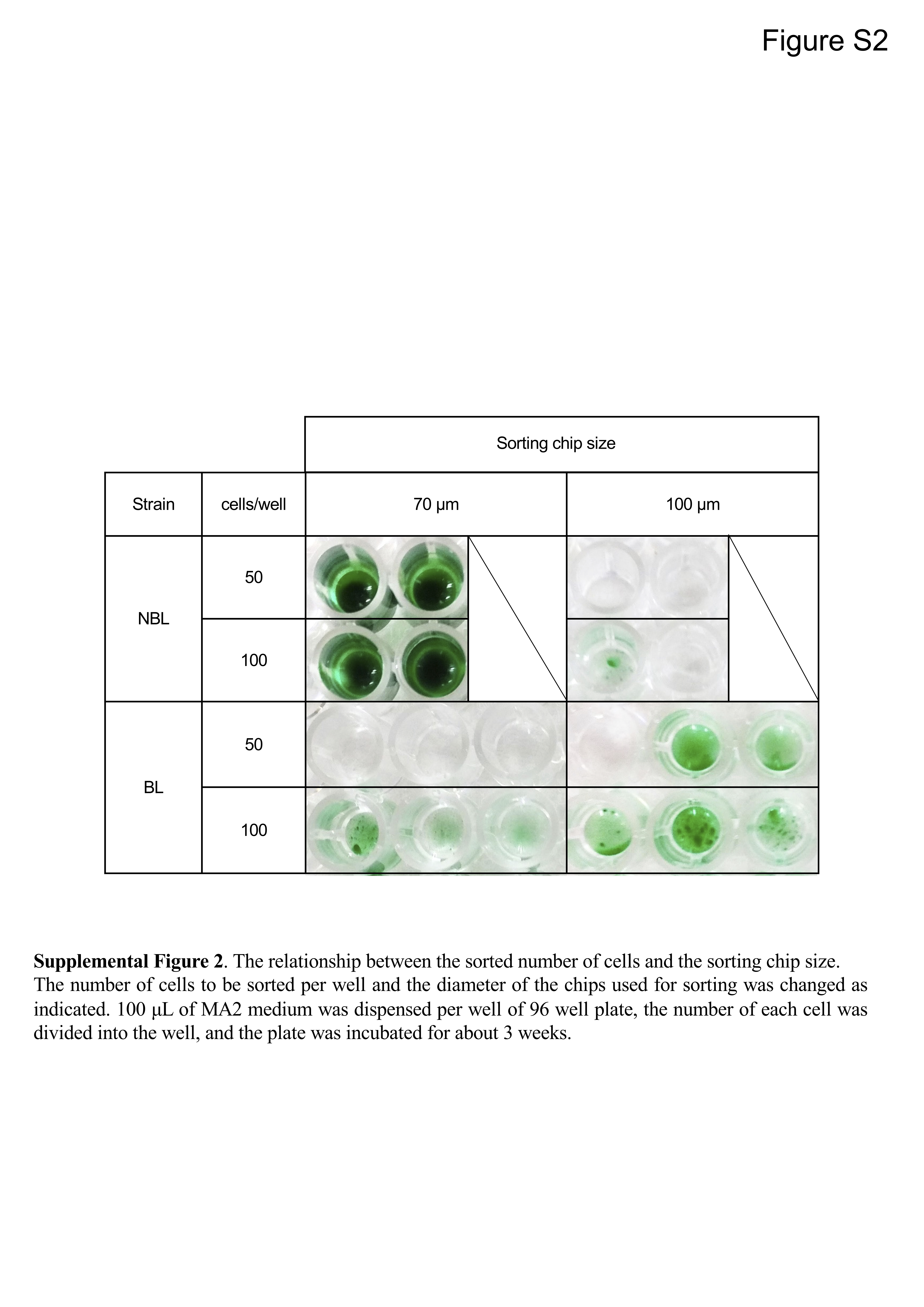

Supplement: Supplementary file 2 [file Image_2.jpeg]
